# Pharmacological inhibition of the lipid phosphatase PTEN ameliorates heart damage and adipose tissue inflammation in stressed rats with metabolic syndrome

**DOI:** 10.14814/phy2.15165

**Published:** 2022-01-10

**Authors:** Sao Ashikawa, Yuki Komatsu, Yumeno Kawai, Kiyoshi Aoyama, Shiho Nakano, Xixi Cui, Misaki Hayakawa, Nanako Sakabe, Nozomi Furukawa, Katsuhide Ikeda, Toyoaki Murohara, Kohzo Nagata

**Affiliations:** ^1^ Pathophysiology Sciences Department of Integrated Health Sciences Nagoya Japan; ^2^ Department of Cardiology Nagoya University Graduate School of Medicine Nagoya Japan

**Keywords:** adipose tissue inflammation, cardiac injury, metabolic syndrome, PTEN, regulatory B cell, stress

## Abstract

Phosphatidylinositol 3‐kinase (PI3K) signaling promotes the differentiation and proliferation of regulatory B (Breg) cells, and the lipid phosphatase phosphatase and tensin homolog deleted on chromosome 10 (PTEN) antagonizes the PI3K–Akt signaling pathway. We previously demonstrated that cardiac Akt activity is increased and that restraint stress exacerbates hypertension and both heart and adipose tissue (AT) inflammation in DS/obese rats, an animal model of metabolic syndrome (MetS). We here examined the effects of restraint stress and pharmacological inhibition of PTEN on heart and AT pathology in such rats. Nine‐week‐old animals were treated with the PTEN inhibitor bisperoxovanadium‐pic [bpV(pic)] or vehicle in the absence or presence of restraint stress for 4 weeks. BpV(pic) treatment had no effect on body weight or fat mass but attenuated hypertension in DS/obese rats subjected to restraint stress. BpV(pic) ameliorated left ventricular (LV) inflammation, fibrosis, and diastolic dysfunction as well as AT inflammation in the stressed rats. Restraint stress reduced myocardial capillary density, and this effect was prevented by bpV(pic). In addition, bpV(pic) increased the proportions of Breg and B‐1 cells as well as reduced those of CD8^+^ T and B‐2 cells in AT of stressed rats. Our results indicate that inhibition of PTEN by bpV(pic) alleviated heart and AT inflammation in stressed rats with MetS. These positive effects of bpV(pic) are likely due, at least in part, to a reduction in blood pressure, an increase in myocardial capillary formation, and an altered distribution of immune cells in fat tissue that result from the activation of PI3K–Akt signaling.

## Graphical abstract

The tumor suppressor phosphatase and tensin homolog deleted on chromosome 10 (PTEN) negatively regulates the phosphatidylinositol 3‐kinase (PI3K)–Akt signaling pathway. Inhibition of PTEN by bpV(pic) did not affect body weight or fat mass but alleviated left ventricular and adipose tissue inflammation in rats with metabolic syndrome associated with stress. These positive effects of bpV(pic) are likely due, at least in part, to a reduction in blood pressure, an increase in myocardial capillary formation, and an altered distribution of immune cells in fat tissue that result from the activation of PI3K–Akt signaling.

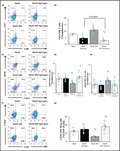

## 1 INTRODUCTION

Metabolic syndrome (MetS) is defined by the development of several conditions that are cardiovascular risk factors and which include hypertension, insulin resistance, obesity, and dyslipidemia. These various conditions are related and have underlying mediators, mechanisms, and pathways in common. Chronic psychological stress increases the risk for obesity and MetS and adversely affects the cardiovascular, immune, and endocrine systems (Uchino et al., 1996). Various levels of the central nervous system contribute to the effects of cytokines on efferent autonomic nerve outflows and consequent modulation of immune responses in the periphery (Kenney & Ganta, 2014).

PTEN (phosphatase and tensin homolog deleted on chromosome 10) is a membrane‐bound lipid phosphatase (Shiojima & Walsh, 2006) that catalyzes the dephosphorylation of phosphatidylinositol (PI) 3,4,5‐trisphosphate to PI 4,5‐bisphosphate and thereby antagonizes signaling by the PI 3‐kinase (PI3K)–Akt pathway. Control of PI3K–Akt signaling by PTEN thus contributes to cellular processes such as cell proliferation, protein synthesis, and glucose metabolism (Vazquez et al., 2006). PTEN negatively regulates PI3Kα and PI3Kγ isoforms in both cardiac myocytes and endothelial cells (Oudit et al., 2004), and PTEN deficiency in the mouse heart increases phosphorylation of Akt, glycogen synthase kinase‐3β, and p70 S6 kinase and thereby leads to cardiac hypertrophy and impairment of cardiac contractility. PTEN also regulates adipose tissue (AT) homeostasis and adipokine secretion (Huang et al., 2019). Studies of transgenic mice have implicated PTEN in the development of AT, although the phenotypes of different models appear inconsistent (Morley et al., 2015; Sanchez‐Gurmaches et al., 2012). Furthermore, although loss or overexpression of PTEN during adipogenesis or in the germ line has revealed a function for PTEN in adipocyte differentiation (Huang et al., 2019), the contribution of PTEN to remodeling of mature AT has remained unclear.

Regulation of PI3K–Akt signaling by PTEN also controls immune function. The differentiation and survival of both B and T lymphocytes are regulated by PI3K–Akt signaling downstream of the corresponding antigen receptors. Mice lacking PTEN specifically in T cells were found to manifest increased numbers of peripheral B cells and CD4^+^ T cells, spontaneous CD4^+^ T cell activation, production of autoantibodies, and hypergammaglobulinemia (Suzuki et al., 2001). Furthermore, PI3K–Akt pathway inhibitors (LY294002 and triciribine) were found to impair the generation of interleukin (IL)‐10–producing regulatory B (Breg) cells in vitro, whereas the number of such cells was increased in mice with B cell‐specific PTEN deficiency (Matsushita et al., 2016).

The DS/obese (DahlS.Z‐*Lepr* ^fa^/*Lepr* ^fa^) rat is a model of MetS that was established as the result of a cross between Dahl salt‐sensitive rats and Zucker rats that harbor a missense mutation in the leptin receptor gene and are not hypertensive (Hattori et al., 2011). When maintained on normal chow, DS/obese rats develop hypertension as well as left ventricular (LV) hypertrophy, fibrosis, and diastolic dysfunction, changes that are accompanied by increased levels of oxidative stress and inflammation (Murase, Hattori, Ohtake, Abe, et al., 2012) as well as of Akt activity (Uchinaka et al., 2018) in the heart. Chronic restraint stress exacerbates hypertension and LV injury in these rats, and this exacerbation is attenuated by administration of the β‐adrenergic antagonist propranolol (Matsuura, Nagasawa, et al., 2015). In addition, restraint stress potentiates heart and AT inflammation in DS/obese rats, again in a manner sensitive to propranolol. On the basis of the hypothesis that inhibition of PTEN might modulate inflammatory immune responses in metabolic disorders exacerbated by chronic stress, we have now tested the effects of pharmacological inhibition of PTEN and restraint stress on heart and AT pathology as well as immune activity in this MetS model.

## 2 METHODS

### 2.1 Animals and protocols

Animal experiments received approval from the Animal Experiment Committee of Nagoya University Graduate School of Medicine (Daiko district, approval nos. 030‐027, 031‐017, 20004, and D210017‐001), and animals were handled in compliance with university guidelines. Eight‐week‐old male inbred DS/obese rats were obtained from Japan SLC and were individually maintained in plastic cages containing bedding. The animals had access to a normal laboratory diet (CE‐2, CLEA Japan) and tap water ad libitum. Beginning at 9 weeks of age, they were injected intraperitoneally with the PTEN inhibitor bisperoxovanadium‐pic [bpV(pic), Sigma‐Aldrich] at 0.2 mg/kg per day (at 0830 h) for 4 weeks, either together with (MetS+RS+bpV(pic) group, *n* = 10) or without (MetS+bpV(pic) group, *n* = 9) exposure to restraint stress in a restraint cage for 2 h each day, as described previously (Matsuura, Nagasawa, et al., 2015). DS/obese rats treated with saline vehicle instead of bpV(pic) and either subjected to restraint stress or not constituted the MetS+RS (*n* = 10) and MetS (*n* = 9) groups, respectively. BpV(pic) binds to the CX5R motif of PTEN, a reactive center common to protein tyrosine phosphatases, and inhibits its activity more specifically than does vanadium (Lai et al., 2007). The median inhibitory concentration of bpV(pic) for PTEN in vitro was found to be 20–40 nM, a value lower than that for other protein tyrosine phosphatases by a factor of 10–100 (Schmid et al., 2004). The experimental animals were monitored weekly for body weight and food and water intakes. Both heart rate and systolic blood pressure (SBP) were also determined weekly in conscious rats by tail‐cuff plethysmography (BP‐98A, Softron; Takatsu et al., 2012). Ketamine (50 mg/kg)–xylazine (10 mg/kg) were injected intraperitoneally at 13 weeks of age to anesthetize the rats for performance of transthoracic echocardiography. After subsequent intraperitoneal administration of sodium pentobarbital (50 mg/kg), the heart and visceral (epididymal and retroperitoneal) and subcutaneous (inguinal) AT were isolated.

### 2.2 Echocardiographic and hemodynamic analyses

Transthoracic echocardiography was performed as described (Kato et al., 2008; Nagata et al., 2002). LV end‐systolic dimension (LVDs), LV end‐diastolic dimension (LVDd), LV posterior wall thickness (LVPWT), and interventricular septum thickness (IVST) were measured by LV short‐axis M‐mode echocardiography with a 12.5‐MHz transducer (Xario SSA‐660A; Toshiba Medical Systems). LV fractional shortening (LVFS), relative wall thickness (RWT), and LV mass were calculated as described (Hattori et al., 2014). LV ejection fraction (LVEF) was calculated from LV end‐diastolic and end‐systolic volumes as determined with the formula of Pombo. Pulsed‐wave Doppler echocardiography was performed to simultaneously record the velocity patterns of LV inflow and outflow for the evaluation of LV functional indexes. LV diastolic function was assessed on the basis of peak flow velocities measured at the mitral level during both rapid filling (*E*) and atrial contraction (*A*) as well as of the *E*/*A* ratio, the deceleration time (DcT), and the isovolumic relaxation time (IRT).

### 2.3 Histology and immunohistochemistry

After fixation for 48 h in phosphate‐buffered saline containing 4% paraformaldehyde, LV and epididymal AT were embedded in paraffin for histological analysis as described (Takatsu et al., 2012). Transverse sections (3 µm) were thus stained with hematoxylin–eosin for the determination of LV myocyte or adipocyte cross‐sectional area, or with Azan Mallory solution for the measurement of LV perivascular and interstitial fibrosis (Takatsu et al., 2012). Immunohistochemical analysis with antibodies to rat CD68 (clone ED1, Chemicon) or those to rat CD31 (clone TLD‐3A12, BD Biosciences) was performed for the evaluation of macrophage infiltration or capillary density, respectively, as previously described (Murase, Hattori, Ohtake, Abe, et al., 2012). Image analysis was conducted with NIH Scion Image (Hattori et al., 2014).

### 2.4 Quantitative RT‐PCR analysis

Total RNA was prepared from LV or epididymal AT as described (Matsuura, Asano, et al., 2015) and was subjected to reverse transcription (RT) with a PrimeScript RT Reagent Kit (Takara). The resultant cDNA was amplified by real‐time polymerase chain reaction (PCR) analysis as performed with SYBR Mix Ex Taq II (Takara), a Thermal Cycler Dice Real Time System II (Takara), and specific primers for atrial natriuretic peptide (ANP; Nagata et al., 2002) brain natriuretic peptide (BNP; Nagata et al., 2002) monocyte chemoattractant protein‐1 (MCP‐1; Nagata et al., 2006) osteopontin (Nagata et al., 2006), tumor necrosis factor‐α (TNF‐α; Murase, Hattori, Ohtake, Abe, et al., 2012) collagen type I or type III (Sakata et al., 2004), vascular endothelial growth factor A (VEGF‐A; Inoue et al., 2003) hypoxia‐inducible factor‐1α (HIF‐1α; Miyachi et al., 1979) endothelial nitric oxide synthase (eNOS; Hudlicka et al., 1992) cyclooxygenase‐2 (COX‐2; Murase, Hattori, Ohtake, Nakashima, et al., 2012) or IL‐10 (Xing et al., 2012). The abundance of mRNAs for target genes was normalized by the amount of glyceraldehyde 3‐phosphate dehydrogenase (GAPDH) mRNA (forward and reverse primers of 5′‐GGGGTGATGCTGGTGCTGAG‐3′ and 5′‐GGTGCAGGATGCATTGCTGAC‐3′, respectively; GenBank accession no. NM_017008.4).

### 2.5 Flow cytometric analysis

Three‐color flow cytometric analysis of lymphocytes in epididymal AT was performed as previously described (Katsuki et al., 2015; Uchinaka et al., 2018) with a FACSCalibur system (BD Biosciences) and the following antibodies (BD Biosciences): fluorescein isothiocyanate‐linked anti‐CD19, allophycocyanin‐linked anti‐CD1d, phycoerythrin‐linked anti‐CD5, allophycocyanin‐linked anti‐B220, allophycocyanin‐linked anti‐CD3, and phycoerythrin‐linked anti‐CD8a. For identification of Breg cells, lymphocytes were defined first with a forward (FSC) and side (SSC) scatter gate. A second (logical) gate was imposed for CD19^+^ B cells. The gated cells were analyzed for CD1d and CD5 expression, and the double‐positive CD19^+^ cells were counted as Breg cells. Likewise, for determination of B‐1, B‐2, and CD8^+^ T cells, lymphocytes were initially gated on the basis of FSC and SSC, and the targeted subsets were identified as cells that were B220^–^ and CD19^+^, B220^+^ and CD19^+^, or CD3^+^ and CD8^+^, respectively.

### 2.6 Statistics

Results are expressed as mean ± SEM values. Data for 13‐week‐old rats were compared among groups by one‐way factorial ANOVA followed by Fisher's multiple comparison test. Time course data were compared by two‐way repeated measures ANOVA. Potential interactive effects of bpV(pic) treatment and restraint stress on various parameters were evaluated by two‐way factorial ANOVA. Statistical significance was defined as a *p* value of <0.05.

## 3 RESULTS

### 3.1 Physiological parameters

**FIGURE 1:**
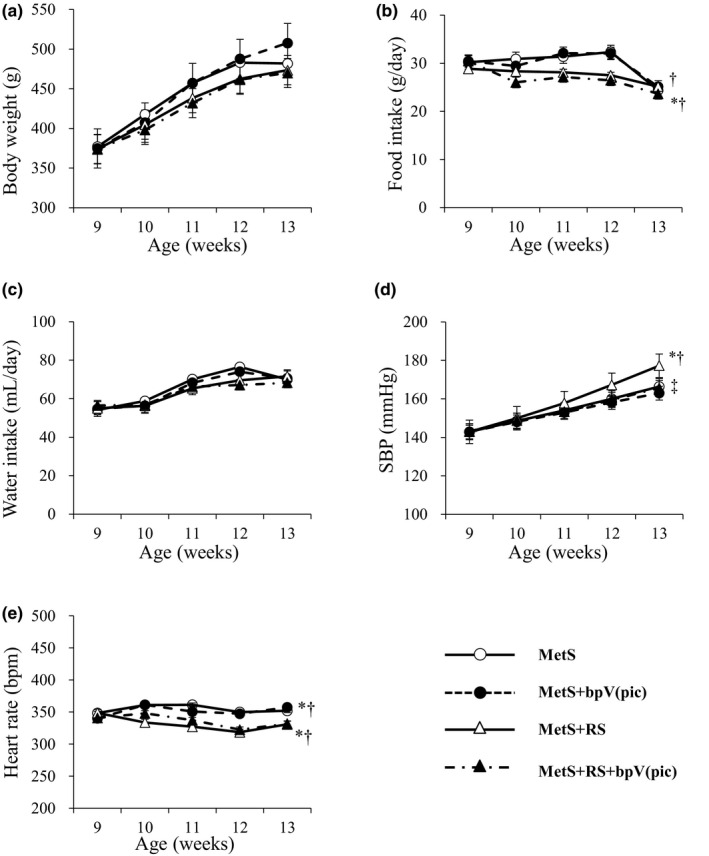
Time courses of body weight (a), food intake (b), water intake (c), SBP (d), and heart rate (e) in rats of the four experimental groups. Data are means ± SEM (*n* = 9, 9, 10, and 10 for MetS, MetS+bpV(pic), MetS+RS, and MetS+RS+bpV(pic) groups, respectively). *p < 0.05 versus MetS, ^†^ *p* < 0.05 versus MetS+bpV(pic), ^‡^ *p* < 0.05 versus MetS+RS (two‐way repeated measures ANOVA)

We determined the effects of bpV(pic) and restraint stress on physiological parameters in DS/obese rats. Neither bpV(pic) nor restraint stress had an effect on body weight or on water intake (Figure 1a,c). Although food intake was not altered by bpV(pic), it was lower in the MetS+RS+bpV(pic) group than in the MetS+bpV(pic) group (Figure 1b). SBP was significantly increased in the MetS+RS group relative to the MetS group at 13 weeks of age, with this increase being attenuated in the MetS+RS+bpV(pic) group (Figure 1d; Table 1). SBP was lower in the MetS+bpV(pic) group than in the MetS group at 13 weeks of age, but the corresponding time courses did not differ. Heart rate was reduced by restraint stress but was not affected by bpV(pic) (Figure 1e; Table 1). Heart weight normalized by tibial length (TL), an index of heart hypertrophy, did not differ significantly among the four groups at 13 weeks (Table 1). LV weight normalized by TL, an index of LV hypertrophy, was smaller in the MetS+RS and MetS+RS+bpV(pic) groups than in the MetS group (Table 1), although no interaction between bpV(pic) and restraint stress was detected for this parameter (Table S1). Epididymal or retroperitoneal fat mass normalized by TL was not affected by bpV(pic) or restraint stress (Table 1). Inguinal fat mass normalized by TL was smaller in the MetS+RS+bpV(pic) group than in the MetS+bpV(pic) group, but again there was no significant interaction between the two treatments (Table S1).

**TABLE 1:** Physiological parameters for rats at 13 weeks of age

Parameter	MetS	MetS+bpV(pic)	MetS+RS	MetS+RS+bpV(pic)
Body weight (g)	481.9 ± 8.0	507.8 ± 8.3	473.5 ± 15.7^†^	470.0 ± 10.9^†^
SBP (mmHg)	166.6 ± 0.6	163.0 ± 0.9*	177.2 ± 1.2* ^,^ ^†^	165.6 ± 1.0^‡^
Heart rate (bpm)	352.0 ± 7.5	357.4 ± 4.3	330.9 ± 6.9* ^,^ ^†^	331.9 ± 8.4^†^
TL (mm)	33.4 ± 0.3	34.3 ± 0.3	34.5 ± 0.4	34.4 ± 0.3
Heart weight/TL (mg/mm)	46.1 ± 0.8	44.5 ± 1.3	43.3 ± 0.9	43.4 ± 1.3
LV weight/TL (mg/mm)	35.3 ± 0.6	33.2 ± 1.0	32.6 ± 0.8*	32.7 ± 0.9*
Epididymal fat weight/TL (mg/mm)	314.1 ± 11.1	354.6 ± 17.5	313.6 ± 18.6	321.2 ± 11.0
Retroperitoneal fat weight/TL (mg/mm)	446.2 ± 13.4	463.6 ± 14.6	439.0 ± 18.7	452.9 ± 11.6
Inguinal fat weight/TL (mg/mm)	1269.2 ± 36.0	1292.8 ± 46.5	1225.6 ± 65.9	1147.0 ± 43.9^†^

Note

Data are means ± SEM (*n* = 9, 9, 10, and 10 for MetS, MetS+bpV(pic), MetS+RS, and MetS+RS+bpV(pic) groups, respectively).

* *p* < 0.05 versus MetS,

^†^ *p* < 0.05 versus MetS+bpV(pic),

^‡^ *p* < 0.05 versus MetS+RS (one‐way factorial ANOVA and Fisher's test).

### 3.2 Heart function

The effects of bpV(pic) and restraint stress on LV morphology and function were evaluated by echocardiography (Table 2). LVDd, LVPWT, IVST, RWT, LV mass, LVFS, and LVEF were similar among the four groups. The E/A ratio was reduced and both IRT and DcT were increased in the MetS+RS group relative to the MetS group, and these changes were attenuated by bpV(pic). These results thus suggested that LV diastolic dysfunction was exacerbated by restraint stress in DS/obese rats, and that this exacerbation was ameliorated by the PTEN inhibitor.

**TABLE 2:** Morphological and functional parameters for the heart of rats at 13 weeks of age

Parameter	MetS	MetS+bpV(pic)	MetS+RS	MetS+RS+bpV(pic)
IVST (mm)	2.37 ± 0.11	2.33 ± 0.09	2.34 ± 0.06	2.29 ± 0.09
LVPWT (mm)	2.37 ± 0.11	2.33 ± 0.09	2.32 ± 0.07	2.32 ± 0.09
LVDd (mm)	7.78 ± 0.24	7.57 ± 0.22	7.75 ± 0.26	7.92 ± 0.23
LVDs (mm)	4.08 ± 0.18	3.92 ± 0.23	3.89 ± 0.15	4.21 ± 0.16
LVFS (%)	47.65 ± 1.54	48.39 ± 2.08	49.87 ± 1.03	47.74 ± 1.16
LVEF (%)	85.22 ± 1.32	85.28 ± 1.52	87.09 ± 0.72	85.19 ± 0.82
LV mass (mg)	1376.3 ± 55.1	1317.2 ± 46.8	1350.6 ± 64.4	1360.5 ± 38.8
RWT	0.62 ± 0.05	0.63 ± 0.04	0.61 ± 0.03	0.59 ± 0.04
E/A	1.55 ± 0.04	1.57 ± 0.07	1.34 ± 0.06* ^,^ ^†^	1.69 ± 0.07^‡^
DcT (ms)	45.71 ± 2.29	42.42 ± 2.75	56.19 ± 1.05* ^,^ ^†^	45.02 ± 0.44^‡^
IRT (ms)	34.11 ± 1.41	34.33 ± 1.42	40.06 ± 1.24* ^,^ ^†^	31.72 ± 2.25^‡^

Note

Data are means ± SEM (*n* = 9, 9, 10, and 10 for MetS, MetS+bpV(pic), MetS+RS, and MetS+RS+bpV(pic) groups, respectively).

* *p* < 0.05 versus MetS,

^†^ *p* < 0.05 versus MetS+bpV(pic),

^‡^ *p* < 0.05 versus MetS+RS (one‐way factorial ANOVA and Fisher's test).

### 3.3 LV tissue pathology

**FIGURE 2:**
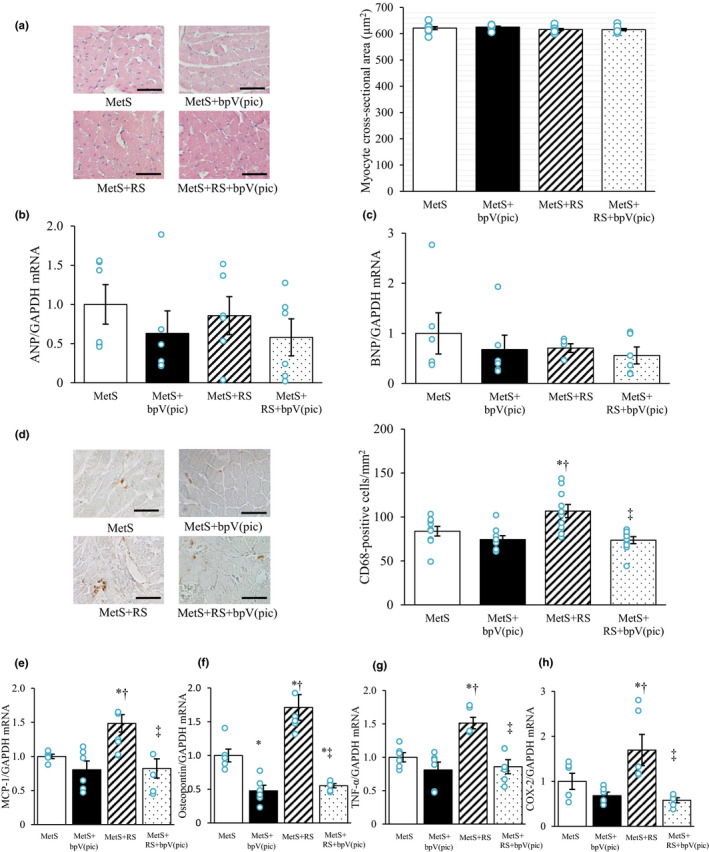
Cardiomyocyte size, fetal‐type cardiac gene expression, macrophage infiltration, and inflammation‐related gene expression in the left ventricle of rats at 13 weeks of age. (a) hematoxylin–eosin staining of transverse sections of the LV myocardium (left; bars, 50 µm) as well as cross‐sectional area of cardiomyocytes measured in such sections (right). (b, c) Relative atrial natriuretic peptide (ANP) (b) and brain natriuretic peptide (BNP) (c) mRNA abundance in left ventricular (LV) tissue. (d) Immunohistochemical staining of CD68 in the left ventricle (left; bars, 50 µm) as well as quantification of CD68^+^ cell density on the basis of such staining (right). (e–h) Relative monocyte chemoattractant protein‐1 (MCP‐1) (e), osteopontin (f), tumor necrosis factor‐α (TNF‐α) (g), and cyclooxygenase‐2 (COX‐2) (h) mRNA abundance in LV tissue. All quantitative data are means ± SEM [*n* = 9, 9, 10, and 9 (a, d) or *n* = 6, 6, 6, and 6 (b, c, and e–h) for MetS, MetS+bpV(pic), MetS+RS, and MetS+RS+bpV(pic) groups, respectively]. *p < 0.05 versus MetS, ^†^ *p* < 0.05 versus MetS+bpV(pic), ^‡^ *p* < 0.05 versus MetS+RS (one‐way factorial ANOVA and Fisher's test)

Histological analysis revealed no significant difference in myocyte cross‐sectional area, and quantitative RT‐PCR analysis did not detect any differences in the expression of ANP or BNP genes in the left ventricle, among the four experimental groups (Figure 2a–c). Immunohistochemical analysis of CD68, a marker for monocytes–macrophages, showed that macrophage infiltration in LV tissue was increased by restraint stress and that this change was attenuated by bpV(pic) (Figure 2d). The amounts of MCP‐1, osteopontin, TNF‐α, and COX‐2 mRNAs in LV tissue were also increased in the MetS+RS group, and such upregulation was suppressed bpV(pic) treatment (Figure 2e–h).

**FIGURE 3:**
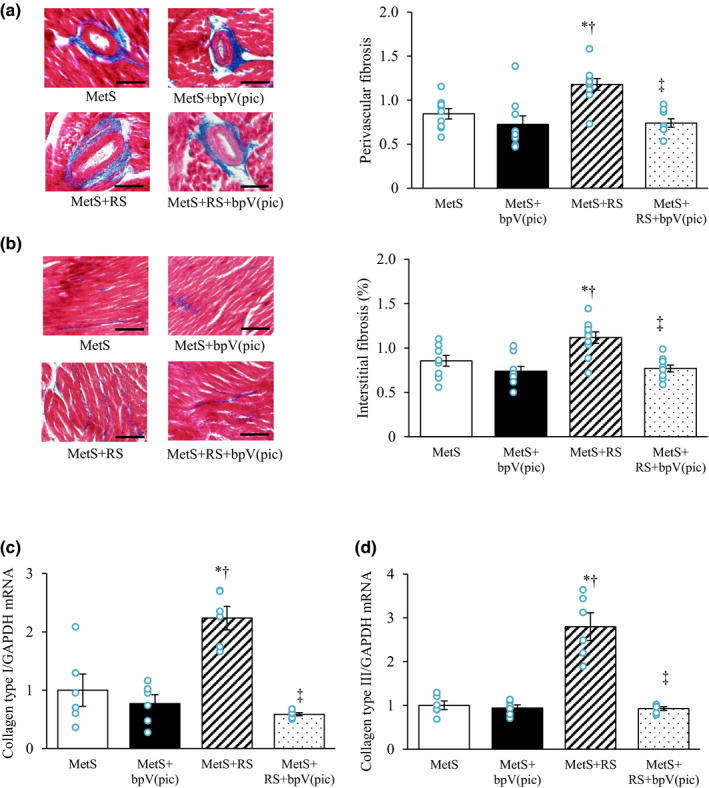
Fibrosis and fibrosis‐related gene expression in the left ventricle of rats at 13 weeks of age. (a, b) Azan Mallory staining of collagen deposition in perivascular (a) and interstitial (b) areas of the left ventricular (LV) myocardium (left; bars, 100 µm) as well as quantification of the relative extents of fibrosis determined from such staining (right). (c, d) Relative collagen type I (c) and type III (d) mRNA abundance in LV tissue. All quantitative data are means ± SEM [*n* = 9, 9, 10, and 10 (a, b) or *n* = 6, 6, 6, and 6 (c, d) for MetS, MetS+bpV(pic), MetS+RS, and MetS+RS+bpV(pic) groups, respectively]. *p < 0.05 versus MetS, †*p* < 0.05 versus MetS+bpV(pic), ‡*p* < 0.05 versus MetS+RS (one‐way factorial ANOVA and Fisher's test)

The extent of perivascular and interstitial fibrosis in the LV myocardium was increased by restraint stress, and this effect was abrogated by bpV(pic) treatment (Figure 3a,b). The abundance of collagen types I and III mRNAs in LV tissue was also increased in the MetS+RS group relative to the MetS group, but such changes were not observed in the MetS+RS+bpV(pic) group (Figure 3c,d).

**FIGURE 4:**
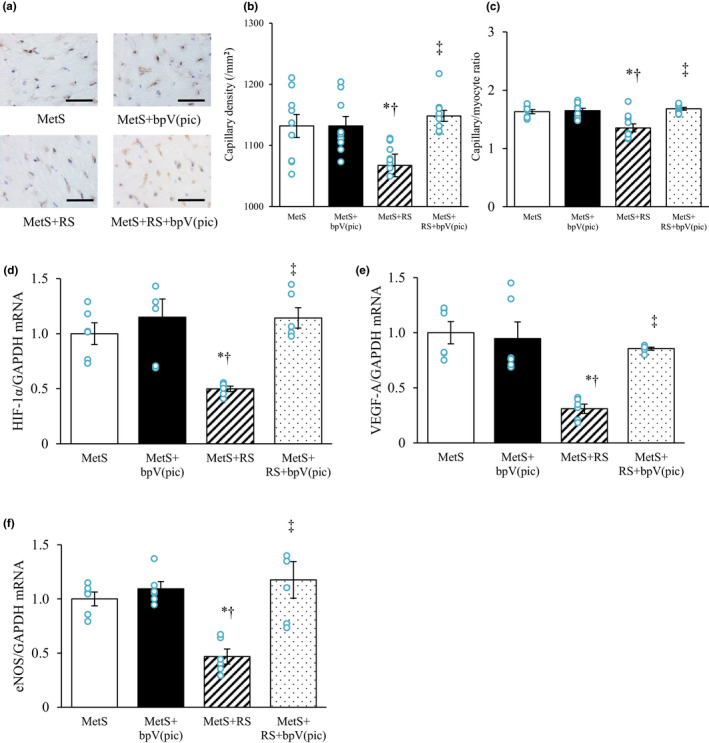
Angiogenesis and expression of pro‐angiogenic genes in the left ventricle of rats at 13 weeks of age. (a–c) Immunohistochemical staining of CD31 in the left ventricle (a) as well as capillary density (b) and the capillary/myocyte ratio (c) determined from such staining. Bars, 50 µm. (d–f) Relative hypoxia‐inducible factor‐1α (HIF‐1α) (d), vascular endothelial growth factor A (VEGF‐A) (e), and endothelial nitric oxide synthase (eNOS) (f) mRNA abundance in LV tissue. All quantitative data are means ± SEM [*n* = 9, 9, 10, and 10 (b, c) or *n* = 6, 6, 6, and 6 (d–f) for MetS, MetS+bpV(pic), MetS+RS, and MetS+RS+bpV(pic) groups, respectively]. *p < 0.05 versus MetS, †*p* < 0.05 versus MetS+bpV(pic), ‡*p* < 0.05 versus MetS+RS (one‐way factorial ANOVA and Fisher's test)

Immunohistochemical analysis of LV tissue for CD31 as a marker of capillary endothelial cells showed that both capillary density and the ratio of the number of coronary capillaries to the number of cardiomyocytes were reduced in the MetS+RS group relative to the MetS group and that these changes were attenuated by treatment with bpV(pic) (Figure 4a–c). Expression of HIF‐1α, VEGF‐A, and eNOS genes in LV tissue was also downregulated in the MetS+RS group relative to the MetS group in a manner sensitive to treatment with the PTEN inhibitor (Figure 4d–f).

### 3.4 AT pathology

**FIGURE 5:**
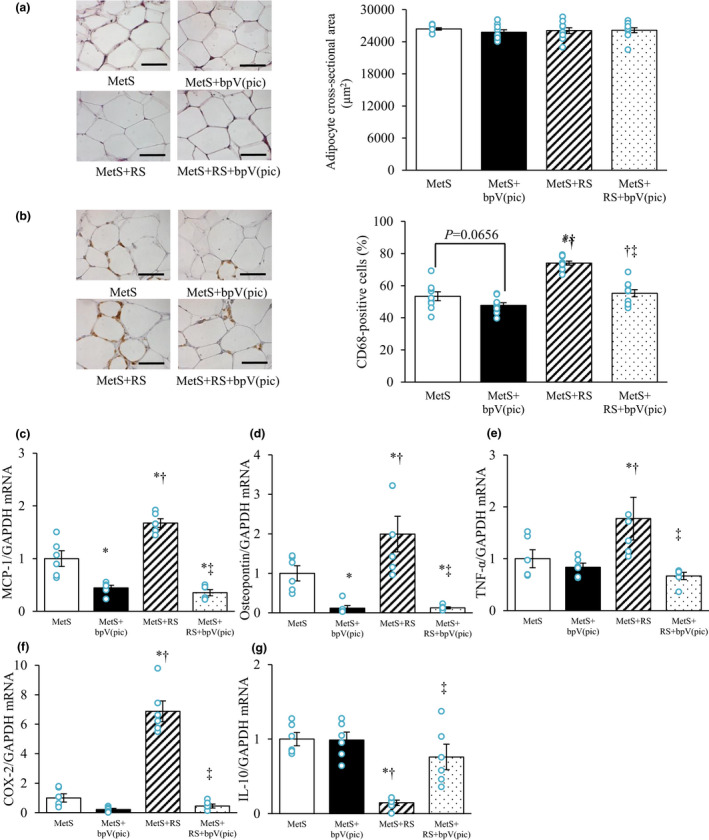
Adipocyte size, macrophage infiltration, and inflammatory gene expression in epididymal adipose tissue (AT) of rats at 13 weeks of age. (a) hematoxylin–eosin staining of AT sections (left; bars, 50 µm) as well as adipocyte cross‐sectional area measured on the basis of such staining (right). (b) Immunohistochemical staining of CD68 in AT (left; bars, 50 µm) as well as the number of nuclei for CD68‐positive cells as a proportion of all nuclei determined from such staining (right). (c–g) Relative monocyte chemoattractant protein‐1 (MCP‐1) (c), osteopontin (d), tumor necrosis factor‐α (TNF‐α) (e), cyclooxygenase‐2 (COX‐2) (f), and IL‐10 (g) mRNA abundance in AT. All quantitative data are means ± SEM [*n* = 9, 9, 10, and 10 (a, b) or *n* = 6, 6, 6, and 6 (c–g) for MetS, MetS+bpV(pic), MetS+RS, and MetS+RS+bpV(pic) groups, respectively]. *p < 0.05 versus MetS, †*p* < 0.05 versus MetS+bpV(pic), ‡*p* < 0.05 versus MetS+RS (one‐way factorial ANOVA and Fisher's test)

The cross‐sectional area of epididymal adipocytes was similar among rats of the four groups (Figure 5a). Immunohistochemical analysis of CD68 revealed that restraint stress increased macrophage infiltration in epididymal AT, and that this effect was attenuated by bpV(pic) treatment (Figure 5b). Expression of MCP‐1 and osteopontin genes in epididymal AT was increased in the MetS+RS group relative to the MetS group, whereas bpV(pic) inhibited the expression of these genes in both MetS and MetS+RS rats (Figure 5c,d). Expression of TNF‐α and COX‐2 genes in epididymal AT was increased by restraint stress in a manner sensitive to inhibition by bpV(pic) treatment (Figure 5e,f). Furthermore, the abundance of IL‐10 mRNA was reduced by restraint stress, and this effect was also prevented by bpV(pic) (Figure 5g).

### 3.5 Immunological analysis

**FIGURE 6:**
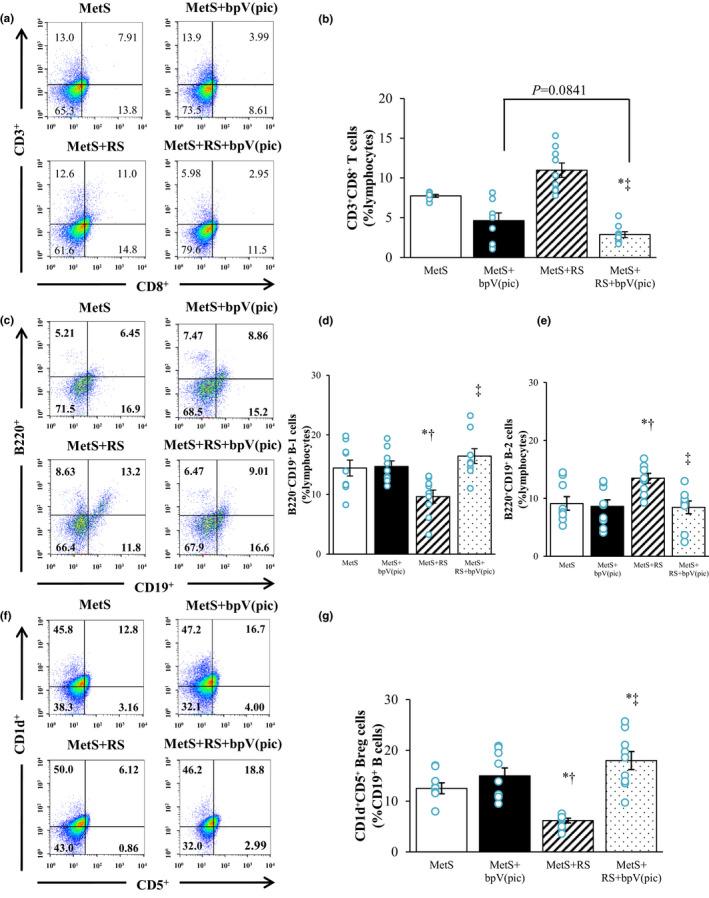
Frequency of CD8^+^ T cells, B‐1 and B‐2 cells, and Breg cells among lymphocytes in epididymal AT of rats at 13 weeks of age. (a, c, and f) Representative flow cytometric dot plots for CD3^+^CD8^+^ T cells among lymphocytes (a), B220^−^CD19^+^ and B220^+^CD19^+^ B cells among lymphocytes (c), and CD1d^+^CD5^+^ cells among CD19^+^ B cells (f). (b, d, e, and g) Percentage of CD3^+^CD8^+^ T cells among lymphocytes (b), B220^–^CD19^+^ B‐1 cells among lymphocytes (D), B220^+^CD19^+^ B‐2 cells among lymphocytes (e), and CD1d^+^CD5^+^ Breg cells among CD19^+^ B cells (g) determined as in (a), (c), and (f). All quantitative data are means ±SEM (*n* = 9, 9, 9, and 9 for MetS, MetS+bpV(pic), MetS+RS, and MetS+RS+bpV(pic) groups, respectively). *p < 0.05 versus MetS, †*p* < 0.05 versus MetS+bpV(pic), ‡*p* < 0.05 versus MetS+RS (one‐way factorial ANOVA and Fisher's test)

Finally, we examined the effects of bpV(pic) and restraint stress on B‐cell and T‐cell fractions in epididymal fat by flow cytometry. The percentage of CD8^+^ T cells among lymphocytes was higher in the MetS+RS group than in the MetS group, and bpV(pic) reduced the proportion of these cells in both MetS and MetS+RS rats (Figure 6a,b). The proportion of B‐1 cells among lymphocytes was reduced by restraint stress, and this effect was prevented by bpV(pic) treatment (Figure 6c,d). The percentage of B‐2 cells among lymphocytes was increased in the MetS+RS group relative to the MetS group, with this effect being abolished by bpV(pic) treatment (Figure 6c,e). The proportion of CD1d^+^CD5^+^ cells among CD19^+^ cells (Breg cells) was reduced by restraint stress, and this change was attenuated by bpV(pic) treatment (Figure 6f,g).

### 3.6 Interaction between bpV(pic) treatment and restraint stress

To examine the influences of bpV(pic) treatment and restraint stress as well as the possible interactions of these factors, we subjected the data obtained in this study to two‐way factorial ANOVA (Table S1). The results of such analysis were essentially consistent with those of one‐way factorial ANOVA are presented in Figures 2, 3, 4, 5, 6 and in Tables 1 and 2.

## 4 DISCUSSION

We have now shown that blockade of PTEN with bpV(pic) attenuated restraint stress‐induced exacerbation of hypertension; LV inflammation, fibrosis, and diastolic dysfunction; and AT inflammation in DS/obese rats. Restraint stress also reduced coronary capillary density and the capillary/cardiomyocyte ratio in this animal model of MetS, with these changes also being attenuated by bpV(pic) treatment. In addition, bpV(pic) increased the proportions of both Breg and B‐1 cells, as well as reduced those of both CD8^+^ T cells and B‐2 cells, in AT of stressed DS/obese rats. In contrast, bpV(pic) did not substantially affect cardiac or AT pathology in nonstressed DS/obese rats.

The production of pro‐inflammatory adipocytokines including TNF‐α and IL‐6 is upregulated, whereas that of anti‐inflammatory adipocytokines such as adiponectin is downregulated in AT in association with obesity, with such changes contributing to inflammatory changes in MetS. Moreover, increased infiltration of macrophages in AT in association with obesity is accompanied by a shift from the anti‐inflammatory M2 to the inflammatory M1 phenotype of these cells (Lumeng et al., 2007). Our present results confirm our previous findings that restraint stress exacerbates AT inflammation regardless of fat mass in DS/obese rats (Matsuura, Nagasawa, et al., 2015). PI3K–Akt signaling attenuates the secretion of inflammatory cytokines (Kai‐lan & Si, 2015), promotes lipid biosynthesis, and inhibits lipolysis (Huang et al., 2018). Our data thus suggest that targeting of PTEN with bpV(pic) attenuated macrophage‐related inflammation in AT through activation of PI3K–Akt signaling in DS/obese rats subjected to restraint stress.

T cells contribute to early stages of AT inflammation in obesity. Obesity has thus been associated with an increased number of CD8^+^ T cells in fat tissue. These cells infiltrate visceral AT prior to the infiltration of M1 macrophages, and they release pro‐inflammatory cytokines including TNF‐α and IL‐6 (Nishimura et al., 2009). Our findings that bpV(pic) reduced the percentage of CD8^+^ T cells and suppressed inflammation in AT of DS/obese rats are consistent with the earlier finding that depletion of CD8^+^ T cells resulted in attenuation of M1 macrophage accumulation in visceral fat of mice with diet‐induced obesity (Nishimura et al., 2009). The proportion of CD11a^high^ cells among CD8^+^ T cells in blood or the stromal vascular compartment of AT was previously shown to be increased in mice with high‐fat diet‐induced obesity compared with lean mice (Jiang et al., 2014). Furthermore, compared with obese wild‐type mice, the number of crownlike structures consisting of macrophages and T cells was reduced in obese CD11a knockout mice. Obese CD11a knockout mice also showed increased phosphorylation of Akt at Ser^473^ in AT. Given that CD11a plays a pivotal role in AT inflammation by contributing to the infiltration and activation of T cells, it is possible that activation of Akt by bpV(pic) was associated with the downregulation of CD11a in T cells of AT of DS/obese rats. The reduction in the proportion of CD8^+^ T cells in AT induced by bpV(pic) treatment may thus have played a role in the inhibition of macrophage‐related inflammatory responses in this tissue of DS/obese rats exposed to restraint stress, with these effects of bpV(pic) likely being dependent on activation of Akt.

B cells participate in immune responses associated with various metabolic conditions—such as obesity, type 2 diabetes, and cardiovascular disease—and they regulate AT inflammation in obesity. B cells are classified into two subsets, B‐1 and B‐2, with B‐1 cells being subdivided into B‐1a and B‐1b cells (Baumgarth, 2011). In visceral AT, B‐1a cells are the predominant source of the anti‐inflammatory cytokine IL‐10 among B cells (Shen et al., 2015). B‐1a cells antagonize the function of B‐2 cells and increase insulin sensitivity through release of IL‐10 and natural immunoglobulin M and consequent modulation of macrophage‐ and T cell‐mediated inflammation (Shen et al., 2015). B‐1b cells specifically attenuate M1 macrophage‐mediated inflammation in visceral AT, and the anti‐inflammatory natural immunoglobulin M antibodies produced by these cells ameliorate diet‐induced glucose intolerance in mice (Harmon et al., 2016). In contrast, B‐2 cells release inflammatory cytokines including TNF‐α, and they exacerbate metabolic disorders (Lund, 2008; Shen et al., 2015). B cells also regulate pro‐inflammatory cytokine production by CD8^+^ and CD4^+^ T cells (Shaikh et al., 2015). We found that bpV(pic) ameliorated the restraint stress‐induced exacerbation of macrophage‐related inflammatory responses in AT of DS/obese rats in association with upregulation of IL‐10 production (likely from the increased proportion of B‐1 cells) and downregulation of inflammatory cytokine production (likely from the reduced proportions of both CD8^+^ T cells and B‐2 cells).

Breg cells produce mostly IL‐10, accounting for most IL‐10 production by B cells, and they suppress T cell‐mediated inflammatory responses (Yanaba et al., 2008). Splenic IL‐10–producing Breg cells manifest the CD5^+^CD19^hi^ phenotype typical of B‐1a cells (Yanaba et al., 2008). In mice with B cell‐specific PTEN deficiency, splenic Breg cells were found within the B‐1 subset and were increased in number in association with activation of PI3K–Akt signaling (Matsushita et al., 2016). We also found that the bpV(pic)‐induced increase in the proportions of both Breg and B‐1 cells in AT of stressed DS/obese rats was associated with increased expression of the IL‐10 gene and a reduced percentage of CD8^+^ T cells. Breg cells suppress the activation of CD8^+^ T cells in fat tissue of obese animals at least in part through IL‐10 secretion, thereby maintaining AT and systemic metabolic homeostasis (Nishimura et al., 2013). Activation of Akt by bpV(pic) may thus have increased the proportions of both Breg and B‐1 cells and thereby reduced that of CD8^+^ T cells in visceral AT of DS/obese rats subjected to restraint stress. Our present findings are also consistent with the previous observation that PI3K–Akt signaling regulates the development of Breg cells (Matsushita et al., 2016).

In the cardiovascular system, macrophage infiltration and inflammatory changes are implicated in fibrosis. We found that treatment with bpV(pic) attenuated the increase in macrophage‐related inflammation in the LV myocardium as well as ameliorated myocardial fibrosis and LV diastolic dysfunction in stressed DS/obese rats. Our results are consistent with previous findings that inflammatory cytokines such as TNF‐α and chemokines such as MCP‐1 contribute to the pathogenesis of myocardial fibrosis and consequent impairment of diastolic function induced by pressure overload (Sun et al., 2007). Moreover, IL‐10–producing B cells were previously found to reduce the proportions of both T helper (Th) 1 and Th17 cells and thereby to ameliorate inflammatory myocardial injury during the early stage of viral myocarditis (Wei et al., 2019). The beneficial cardiac effects of bpV(pic) in DS/obese rats subjected to restraint stress are thus likely due at least in part to the associated reduction in SBP as well as to the increase in the proportions of IL‐10–producing B cell subsets (Breg and B‐1 cells), decreases in those of both CD8^+^ T cells and B‐2 cells, and downregulation of inflammatory cytokine expression in AT. Although bpV(pic) reduced the proportion of CD8^+^ T cells in AT of nonstressed DS/obese rats, it had no effect on those of Breg or B‐1 cells, possibly explaining in part why the drug did not manifest a cardioprotective effect in these animals.

The PI3K–Akt signaling pathway regulates cardiac growth and coronary angiogenesis (Shiojima & Walsh, 2006). Coronary capillary number and cardiomyocyte size are thought to become mismatched, giving rise to myocardial hypoxia, during progression of cardiac hypertrophy (Tomanek, 1990). A reduction in the extent of coronary angiogenesis on a background of load‐induced cardiac growth may therefore give rise to an imbalance between heart growth and coronary angiogenesis, with this imbalance, rather than the extent of hypertrophy per se, being largely responsible for the transition from physiological to pathological hypertrophy (Miyachi et al., 1979). Furthermore, insufficient angiogenesis is associated with the development of hypertension (Toblli et al., 2004), possibly as a result of downregulation of nitric oxide (NO) production by endothelial cells due to attenuation of VEGF function(Robinson et al., 2010). Our finding that expression of angiogenesis‐related genes such as those for HIF‐1α, VEGF‐A, and eNOS in the heart of stressed DS/obese rats was increased by treatment with bpV(pic) is consistent with the previous findings that activation of PI3K–Akt signaling increases VEGF secretion by both HIF‐1–dependent and –independent mechanisms as well as regulates angiogenesis through modulation of the production of NO and angiopoietins (Karar & Maity, 2011). In addition, increased NO production likely contributes to suppression of LV inflammation. Nuclear factor‐κB (NF‐κB) is a central transcription factor in inflammatory responses. Under normal conditions, NF‐κB is maintained inactive in the cytosol as a result of its interaction with inhibitory IκB proteins (Nandipati et al., 2017). NO induces and stabilizes IκBα, thereby inhibiting the nuclear translocation of NF‐κB (Peng et al., 1995). The beneficial cardiac effects of bpV(pic) in DS/obese rats exposed to restraint stress might therefore be explained in part by the reduction in blood pressure, increased coronary angiogenesis, and a direct anti‐inflammatory effect mediated by activation of PI3K–Akt–HIF‐1α–VEGF–eNOS/NO signaling. The absence of an antihypertrophic effect of bpV(pic) in stressed DS/obese rats is likely due to compensation of the growth‐retarding effect of load reduction by the growth‐promoting effect of Akt activation.

This study has some limitations. First, we analyzed both LV and epididymal AT but not other organs in the animals. We need to be careful about the possible effects of bpV(pic) on other organs because systemic inflammation that originates from excess visceral AT is a common feature of MetS. Future studies are warranted to determine the systemic effects of bpV(pic). Second, we did not examine the bone marrow. Since both cells of the monocyte‐macrophage lineage and B cells are generated in the bone marrow after birth, flow cytometric analysis of hematopoietic (myeloid or lymphoid) populations in the bone marrow may also be useful in rats (Francis et al., 2019) Future studies may be considered to determine the effects of bpV(pic) on immune cells including macrophages and B cells in the bone marrow. Finally, although our data support the notion that bpV(pic) induces activation of Akt through inhibition of PTEN, we have no data for Akt activity with or without bpV(pic) in LV and fat tissue of stressed and nonstressed animals. Further studies are required.

In conclusion, inhibition of PTEN by bpV(pic) ameliorated LV and AT pathology in DS/obese rats subjected to restraint stress. Such amelioration was likely mediated by two key mechanisms: (Baumgarth, 2011) reduced blood pressure and promotion of coronary capillary formation via activation of a PI3K–Akt–HIF‐1α–VEGF–eNOS/NO pathway, resulting in suppression of LV inflammation, fibrosis, and diastolic dysfunction and (Francis et al., 2019) alteration of the distribution of immune cells in AT, without a change in fat mass, induced by activation of PI3K–Akt signaling, and resulting in attenuation of AT inflammation. Further studies are warranted to uncover the precise molecular mechanisms underpinning these actions of PTEN inhibition in MetS associated with stress.

## DISCLOSURE

None.

## Supporting information



Table S1
