# Supplementary material for: Pharmacological inhibition of the lipid phosphatase PTEN ameliorates heart damage and adipose tissue inflammation in stressed rats with metabolic syndrome
Source: Physiol Rep. 2022 Jan 10;10(1):e15165. doi: 10.14814/phy2.15165 (PMC8744130; doi:10.14814/phy2.15165)
Supplement: Supplementary file 1 — Table S1 [file PHY2-10-e15165-s001.docx]

**Supplemental Table 1.** Results of Two-Way Factorial ANOVA for the Effects of BpV(pic) Treatment and Restraint Stress in DS/Obese Rats of the Four Experimental Groups at 13 Weeks of Age

| Parameter | Influence of bpV(pic)  treatment | | Influence of restraint stress | | Interaction | |
| --- | --- | --- | --- | --- | --- | --- |
|  | *F* value | *P* value | *F* value | *P* value | *F* value | *P* value |
| Body weight (g) | 0.952 | 0.3360 | 4.050 | 0.0521 | 1.641 | 0.2088 |
| SBP (mmHg) | 63.150 | <0.0001 | 47.485 | <0.0001 | 17.219 | 0.0002 |
| Heart rate (bpm) | 0.210 | 0.6498 | 11.014 | 0.0022 | 0.098 | 0.7560 |
| Tibial length (mm) | 0.057 | 0.8130 | 0.254 | 0.6173 | 0.001 | 0.9766 |
| Heart weight/tibial length (mg/mm) | 0.446 | 0.5088 | 3.307 | 0.0778 | 0.635 | 0.4311 |
| LV weight/tibial length (mg/mm) | 1.487 | 0.2311 | 3.658 | 0.0643 | 1.705 | 0.2005 |
| Epididymal fat weight/tibial length (mg/mm) | 2.553 | 0.1194 | 1.263 | 0.2690 | 1.191 | 0.2828 |
| Retroperitoneal fat weight/tibial length (mg/mm) | 1.104 | 0.3008 | 0.360 | 0.5522 | 0.013 | 0.9087 |
| Inguinal fat weight/tibial length (mg/mm) | 0.300 | 0.5873 | 3.554 | 0.0680 | 1.032 | 0.3168 |
| IVST (mm) | 0.235 | 0.6311 | 0.097 | 0.7580 | 0.004 | 0.9478 |
| LVPWT (mm) | 0.049 | 0.8257 | 0.103 | 0.7502 | 0.027 | 0.8715 |
| LVDd (mm) | 0.006 | 0.9399 | 0.445 | 0.5093 | 0.639 | 0.4296 |
| LVDs (mm) | 0.210 | 0.6494 | 0.086 | 0.7712 | 1.715 | 0.1991 |
| LVFS (%) | 0.221 | 0.6413 | 0.286 | 0.5961 | 0.945 | 0.3378 |
| LVEF (%) | 0.687 | 0.4129 | 0.639 | 0.4297 | 0.776 | 0.3844 |
| LV mass (mg) | 0.220 | 0.6418 | 0.028 | 0.8681 | 0.434 | 0.5147 |
| RWT | 0.012 | 0.9144 | 0.349 | 0.5589 | 0.133 | 0.7178 |
| E/A | 8.021 | 0.0077 | 0.485 | 0.4911 | 6.686 | 0.0142 |
| DcT (ms) | 16.177 | 0.0003 | 13.232 | 0.0009 | 4.806 | 0.0353 |
| IRT (ms) | 6.004 | 0.0196 | 1.014 | 0.3211 | 6.680 | 0.0142 |
| LV myocyte cross-sectional area (µm^2^) | 0.133 | 0.7173 | 3.001 | 0.0923 | 0.260 | 0.6137 |
| ANP/GAPDH mRNA in LV tissue | 1.921 | 0.1810 | 0.173 | 0.6820 | 0.041 | 0.8419 |
| BNP/GAPDH mRNA in LV tissue | 0.922 | 0.3484 | 0.721 | 0.4060 | 0.127 | 0.7250 |
| CD68-positive cells in LV tissue (/mm^2^) | 14.850 | 0.0005 | 3.995 | 0.0537 | 4.559 | 0.0400 |
| MCP-1/GAPDH mRNA in LV tissue | 16.313 | 0.0006 | 5.666 | 0.0274 | 4.795 | 0.0406 |
| Osteopontin/GAPDH mRNA in LV tissue | 64.100 | <0.0001 | 13.998 | 0.0013 | 9.166 | 0.0066 |
| TNF-α/GAPDH mRNA in LV tissue | 22.765 | 0.0001 | 10.024 | 0.0049 | 6.905 | 0.0161 |
| COX-2/GAPDH mRNA in LV tissue | 15.352 | 0.0009 | 2.632 | 0.1204 | 4.765 | 0.0411 |
| Perivascular fibrosis in LV tissue | 16.324 | 0.0003 | 6.417 | 0.0161 | 5.392 | 0.0264 |
| Interstitial fibrosis in LV tissue (%) | 17.528 | 0.0002 | 6.935 | 0.0126 | 4.314 | 0.0454 |
| Collagen type I/GAPDH mRNA in LV tissue | 29.942 | <0.0001 | 9.409 | 0.0061 | 17.049 | 0.0005 |
| Collagen type Ⅲ/GAPDH mRNA in LV tissue | 38.332 | <0.0001 | 32.651 | <0.0001 | 33.571 | <0.0001 |
| Capillary density in LV tissue (/mm^2^) | 6.560 | 0.0150 | 2.318 | 0.1371 | 6.503 | 0.0154 |
| Capillary/myocyte ratio in LV tissue | 12.224 | 0.0013 | 6.304 | 0.0170 | 10.638 | 0.0025 |
| HIF-1α/GAPDH mRNA in LV tissue | 16.477 | 0.0006 | 6.753 | 0.0172 | 6.378 | 0.0201 |
| VEGF-A/GAPDH mRNA in LV tissue | 8.269 | 0.0093 | 20.882 | 0.0002 | 12.211 | 0.0023 |
|  |  |  |  |  |  |  |
| eNOS/GAPDH mRNA in LV tissue | 18.358 | 0.0004 | 5.788 | 0.0259 | 10.757 | 0.0037 |
| Adipocyte cross-sectional area (µm^2^) | 0.369 | 0.5476 | 0.004 | 0.9484 | 0.599 | 0.4443 |
| CD68-positive cells in adipose tissue (%) | 35.565 | <0.0001 | 47.366 | <0.0001 | 10.259 | 0.0030 |
| MCP-1/GAPDH mRNA in adipose tissue | 121.705 | <0.0001 | 11.820 | 0.0026 | 20.229 | 0.0002 |
| Osteopontin/GAPDH mRNA in adipose tissue | 36.919 | <0.0001 | 4.910 | 0.0385 | 4.732 | 0.0418 |
| TNF-α/GAPDH mRNA in adipose tissue | 9.175 | 0.0066 | 2.083 | 0.1645 | 5.038 | 0.0363 |
| COX-2/GAPDH mRNA in adipose tissue | 103.557 | <0.0001 | 74.385 | <0.0001 | 63.498 | <0.0001 |
| IL-10/GAPDH mRNA in adipose tissue | 8.650 | 0.0081 | 28.005 | <0.0001 | 9.449 | 0.0060 |
| CD3⁺CD8⁺ T cells in adipose tissue (%lymphocytes) | 65.147 | <0.0001 | 1.134 | 0.2949 | 12.862 | 0.0011 |
| B220⁻CD19⁺ B-1 cells in adipose tissue (%lymphocytes) | 9.369 | 0.0044 | 1.762 | 0.1938 | 7.989 | 0.0080 |
| B220⁺CD19⁺ B-2 cells in adipose tissue (%lymphocytes) | 6.670 | 0.0146 | 3.786 | 0.0605 | 4.445 | 0.0429 |
| CD1d⁺CD5⁺ Breg cells in adipose tissue (%CD19^+^ B cells) | 29.332 | <0.0001 | 1.605 | 0.2143 | 12.626 | 0.0012 |
|  |  |  |  |  |  |  |
